# Identification of ubiquitination-related genes in human glioma as indicators of patient prognosis

**DOI:** 10.1371/journal.pone.0250239

**Published:** 2021-04-29

**Authors:** Lei Wang, Yuelin Liu, Chengmin Xuan, Yong Liu, Hengliang Shi, Yong Gao

**Affiliations:** 1 Department of Neurosurgery, Brain Hospital, Affiliated Hospital of Xuzhou Medical University, Xuzhou, Jiangsu, P.R. China; 2 Department of Clinical Medicine, Xuzhou Medical University, Xuzhou, Jiangsu, P.R. China; 3 Medical Section, Xuzhou Children’s Hospital of Xuzhou Medical University, Xuzhou, Jiangsu, P.R. China; 4 Central Laboratory, Affiliated Hospital of Xuzhou Medical University, Xuzhou, Jiangsu, P.R. China; 5 Department of Orthopaedics, Xuzhou Children’s Hospital of Xuzhou Medical University, Xuzhou, Jiangsu, P.R. China; All India Institute of Medical Sciences, INDIA

## Abstract

Ubiquitination is a dynamic and reversible process of a specific modification of target proteins catalyzed by a series of ubiquitination enzymes. Because of the extensive range of substrates, ubiquitination plays a crucial role in the localization, metabolism, regulation, and degradation of proteins. Although the treatment of glioma has been improved, the survival rate of patients is still not satisfactory. Therefore, we explore the role of ubiquitin proteasome in glioma. Survival-related ubiquitination related genes (URGs) were obtained through analysis of the Genotype-Tissue Expression (GTEx) and the Cancer Genome Atlas (TCGA). Cox analysis was performed to construct risk model. The accuracy of risk model is verified by survival, Receiver operating characteristic (ROC) and Cox analysis. We obtained 36 differentially expressed URGs and found that 25 URGs were related to patient prognosis. We used the 25 URGs to construct a model containing 8 URGs to predict glioma patient risk by Cox analysis. ROC showed that the accuracy rate of this model is 85.3%. Cox analysis found that this model can be used as an independent prognostic factor. We also found that this model is related to molecular typing markers. Patients in the high-risk group were enriched in multiple tumor-related signaling pathways. In addition, we predicted TFs that may regulate the risk model URGs and found that the risk model is related to B cells, CD4 T cells, and neutrophils.

## Introduction

The ubiquitination pathway is a highly conserved post-translational modification process regulated by a series of enzymes [1, 2]. It plays a leading role in the degradation of most proteins in eukaryotic cells. Owing to the wide range of substrates that are targeted by ubiquitination, the ubiquitination pathway participates in multiple cellular events, some of which underlie cancer development [3, 4]. The 76-amino acid ubiquitin is activated by the E1 ubiquitin activating enzyme. Activated ubiquitin binds to the E2 ubiquitin binding enzyme through a transthioesterification reaction, and the E3 ubiquitin ligase, which interacts with substrates, binds to the E2 and transfers ubiquitin to the target substrate. The above process requires the catalysis of more than 100 E3 ligases to give substrate specificity [3, 5, 6]. Deubiquitinating enzymes can uncouple ubiquitin or polyubiquitin chains from target proteins, thereby playing the opposite role in negatively regulating the ubiquitin-mediated degradation of proteins [5, 7].

Ubiquitination has been the subject of increasing attention, as research has shown that ubiquitination dysfunction is related to the occurrence and development of many cancers [8–11]. For example, many E3 ubiquitin ligases are closely related to cell cycle progression, p53 activation, DNA damage repair, and tumor cell invasion and migration in tumors [8, 12–14]. In addition, owing to the extensive substrates of the ubiquitination pathway and the regulation of target protein levels, rather than biological activity, the ubiquitination pathway has become a promising therapeutic direction for cancer. Regulating E3 ubiquitin ligases or deubiquitination enzymes may be a strategy to regulate key proteins involved in cancer development, thereby treating cancer [15, 16]. At present, several drugs that regulate the ubiquitination pathway are used clinically [17, 18].

Considering that the ubiquitin proteasome plays a major role in protein degradation and is closely related to tumors, we decided to analyze the role of all known URGs in glioma. By consulting relevant information, we obtained the ubiquitinated gene set, which includes 929 ubiquitin ligase genes and 95 deubiquitination-related genes [19]. By analyzing a comprehensive list of 1024 URGs in 697 patients with glioma, we maximized the chance of scientific and clinical discovery. We found not only to discover the regulatory mechanism of ubiquitin proteasome in gliomas, but also we provide new evaluation criteria for the prognosis of gliomas and new directions for drug development.

## Materials and methods

### Patient samples

MRNA files of glioma and control specimens were obtained from UCSC (https://xena.ucsc.edu/). The GTEx dataset includes 1152 brain tissues that were healthy while alive. The TCGA dataset contains 5 non-tumor brain tissues and 697 glioma tissues, which included 221 World Health Organization (WHO) grade II gliomas, 244 WHO grade III gliomas, 166 grade WHO IV gliomas, and 66 without tissue grade (Table 1). GSE108474 (https://www.ncbi.nlm.nih.gov/geo/query/acc.cgi) contains 671 samples, which included 21 cases of non-tumor tissue, 2 WHO grade I gliomas, 99 WHO grade II gliomas, 84 WHO grade III gliomas, 134 grade WHO IV gliomas, and 331 without tissue grade (Table 1). The WHO classification system was used according to our previous descriptions [6, 20]. All data comes from public databases (GTEx, TCGA, GSE108474) and is completely anonymous. The ethics committee of Xuzhou Children’s Hospital approved the study protocol.

**Table 1:** Comprehensive demographic information of the patients.

	TCGA		GSE108474			GTEx	
1	2		2		1	2	
Gender					Gender		
Male	368	52.42	221	32.94	Male	792	68.75
Female	268	38.18	126	18.78	Female	360	31.25
Age (years)					Body site		
≤65	549	78.21			Amygdala	69	5.99
>65	87	12.39			Anterior cingulate	83	7.20
WHO grade					Basal ganglia	294	25.52
Non-tumor	5	0.71	21	3.13	Cerebellum	216	18.75
Grade I			2	0.30	Cortex	207	17.97
Grade II	221	31.48	99	14.75	Hippocampus	84	7.29
Grade III	244	34.76	84	12.52	Spinal cord	82	7.12
Grade IV	166	16.52	134	19.97	Substantia nigra	60	5.21
Survival status							
Alive	432	61.54	82	12.22			
Deceased	204	29.06	313	46.65			

WHO = World Health Organization. 1 = Patient characteristics, 2 = No. patients (%). Blank space indicates that the corresponding database has no relevant information.

### Identification of survival-related ubiquitination genes

By referring to the literature, we obtained ubiquitination-related genes (URGs), including 929 ubiquitin ligase genes and 95 deubiquitination enzyme genes (S1 Table). The authors obtained the ubiquitination gene set through the Ubiquitin and Ubiquitin-like Conjugation Database (UUCD, http://uucd.biocuckoo.org/) [19]. UUCD uses the ubiquitination protein sequence and hidden Markov model (HMM) calculation methods to predict other possible ubiquitination enzymes and adaptors [19]. The ubiquitination gene set includes a total of 929 genes, including E1 (8 genes, predicted 1), E2 (39 genes, predicted 2) and E3 (882 genes, predicted 368) [19]. The author obtained 95 deubiquitination genes by consulting the literature [19]. Using the limma package of R software (https://www.r-project.org/), we analyzed the differential expression of non-tumor brain tissue and glioma tissue in the GTEx and TCGA databases, and then we only retained the URGs in the differentially expressed genes (Table 2). URGs satisfying FDR<0.05 and log2 |fold change|> 1 are considered statistically significant. Using the R software survival package, we discovered URGs related to the prognosis of patients. A p-value less than 0.05 was considered statistically significant.

**Table 2:** The *p* value and AUC value of 36 differentially expressed URGs.

Survival curve				ROC curve			
Gene	p value	Gene	p value	Gene	AUC	Gene	AUC
**CDC20**	0	KLHL1	0.002	*CDC26*	0.188	*TRIM54*	0.475
TRIM17	0	WDR72	0.006	*TRIM17*	0.213	*USP50*	0.521
**UBE2C**	0	DNAI1	0.018	*CORO6*	0.214	NEDD4	0.542
**WDR62**	1.11E-16	RNF148	0.025	RFPL4B	0.233	KLHL6	0.542
CORO6	3.33E-16	CDRT1	0.060	WDR87	0.238	*SAG*	0.559
**HOXB4**	3.33E-16	KLHL6	0.098	USP6	0.290	*TRIM74*	0.568
**DTL**	2.22E-15	DCAF4L2	0.127	*ASB4*	0.295	*RNF133*	0.570
CDC26	6.33E-15	TRIM74	0.139	*TRIM55*	0.308	UHRF1	0.575
WDR87	1.86E-13	UBE2NL	0.144	*TRIM72*	0.333	DCAF4L2	0.589
RFPL4B	5.40E-12	RNF133	0.144	*DNAI1*	0.374	WDR72	0.618
**TRIM38**	3.23E-10	USP50	0.183	*KLHL1*	0.375	*RNF148*	0.618
USP6	9.69E-09	SAG	0.193	*RNF212*	0.387	CCIN	0.637
TRIM55	1.42E-08	NEDD4	0.222	KLHL31	0.403	**TRIM38**	0.720
ASB4	1.07E-07	UHRF1	0.264	*CDRT1*	0.424	**HOXB4**	0.796
CCIN	3.97E-06	RNF151	0.576	UBE2NL	0.428	**DTL**	0.801
RNF212	7.22E-06	TRIM31	0.621	*SH3RF2*	0.431	**WDR62**	0.835
TRIM72	1.40E-05	SH3RF2	0.676	*TRIM31*	0.455	**UBE2C**	0.839
KLHL31	0.001	TRIM54	0.784	*RNF151*	0.470	**CDC20**	0.866

The bold genes are those with Survival curve p value less than 0.05 and ROC curve with AUC value greater than 0.7. The italic genes in ROC are down-regulated genes, and the rest are up-regulated genes.

### Survival curve analysis and receiver operating characteristic (ROC) curve analysis

We analyzed the relationship between differentially expressed URGs and the risk model with patient prognosis using the survival package of R software. The results were analyzed by survival curves. The survival ROC package of R software was used to analyze the accuracy of URGs and the risk model in predicting patient prognosis. P<0.05 (for survival curves) and AUC>0.7 (for ROC curves) were considered to indicate statistical significance.

### Univariate and multivariate Cox analysis

To analyze whether the URGs and the risk model were prognostic factors for glioma patients, we analyzed the relationship between the individual factors and the survival of patients by univariate Cox analysis. We used multivariate Cox to analyze whether the factors and model can be used as prognostic factors for patients. The analyses were performed using R software.

### Risk model of URGs and patient risk level calculations

We analyzed the correlation between differentially expressed URGs and survival through R software, and obtained URGs related to survival. Then, we performed multivariate Cox regression analysis on survival-related URGs, excluded URGs that could complement survival relationships, and constructed an optimal risk model for predicting patient prognosis. After obtaining the risk model, we multiplied the risk model gene expression level of each patient by the model coefficient to obtain the risk score of each patient. We divided patents into high- and low-risk groups according to the median value of the risk score.

### Gene Set Enrichment Analysis (GSEA) of risk model-enriched KEGG pathways

We used GSEA (http://software.broadinstitute.org/gsea/index.jsp) to analyze the risk model-enriched KEGG signaling pathways. We calculate the risk value of each patient according to the risk model formula. According to the median risk value, patients were divided into high and low risk groups. Then the KEGG signaling pathway enriched in the high- and low- risk groups was analyzed by GSEA.

### Correlation analysis between risk model and clinical markers

To explore the relationship between the risk model and common clinical traits, we analyzed the relationship between the risk model and these common clinical traits, such as ATRX status, Chr 19/20 co-gain, Chr 7 gain/Chr 10 loss, MGMT promoter status, 1P/19q co-deletion, IDH status, mutation count, histology, gender, grade, age and fustat (state of existence) [20]. We found that the risk model is related to all clinical traits except gender. **p<0.01, ***p<0.001.

### Analysis of transcription factors (TFs) regulating URGs in the risk model

Cistrome Cancer contains a regulatory link between TFs and the transcriptome, which is constructed by TCGA tumor molecules, 23,000 ChIP-seq and chromatin [21]. The Cistrome Cancer database contains 318 TFs. We used clinically relevant TFs to construct regulatory networks of TFs and URGs. A P-value<0.001 was considered statistically significant.

### Analysis of the correlation between the risk model and immune cells

We used TIMER (Tumor Immune Estimation Resource) to analyze the numbers of six subtypes of tumor-infiltrating immune cells, including B cells, CD4 T cells, CD8T cells, dendritic cells, macrophages, and neutrophils of tumor-infiltrating immune cells, in 10,897 samples from 32 cancers of TCGA [22]. We downloaded the level of glioma immune infiltration by TIMER (https://cistrome.shinyapps.io/timer/) and analyzed the relationship between the risk score obtained and immune cell infiltration.

### Statistical analysis

R software was used for statistical analyses. The differentially expressed URGs were obtained by Wilcoxon test. Statistical significance was defined as a two-tailed *P* value < 0.05. GSEA was performed using GSEA software, where |NES (normalized enrichment score)| > 1, NOM p-value < 0.05, and FDR q-value < 0.25 were considered to indicate statistical significance.

## Results

### Identification of differentially expressed URGs and survival-related ubiquitination genes

**Fig 1:**
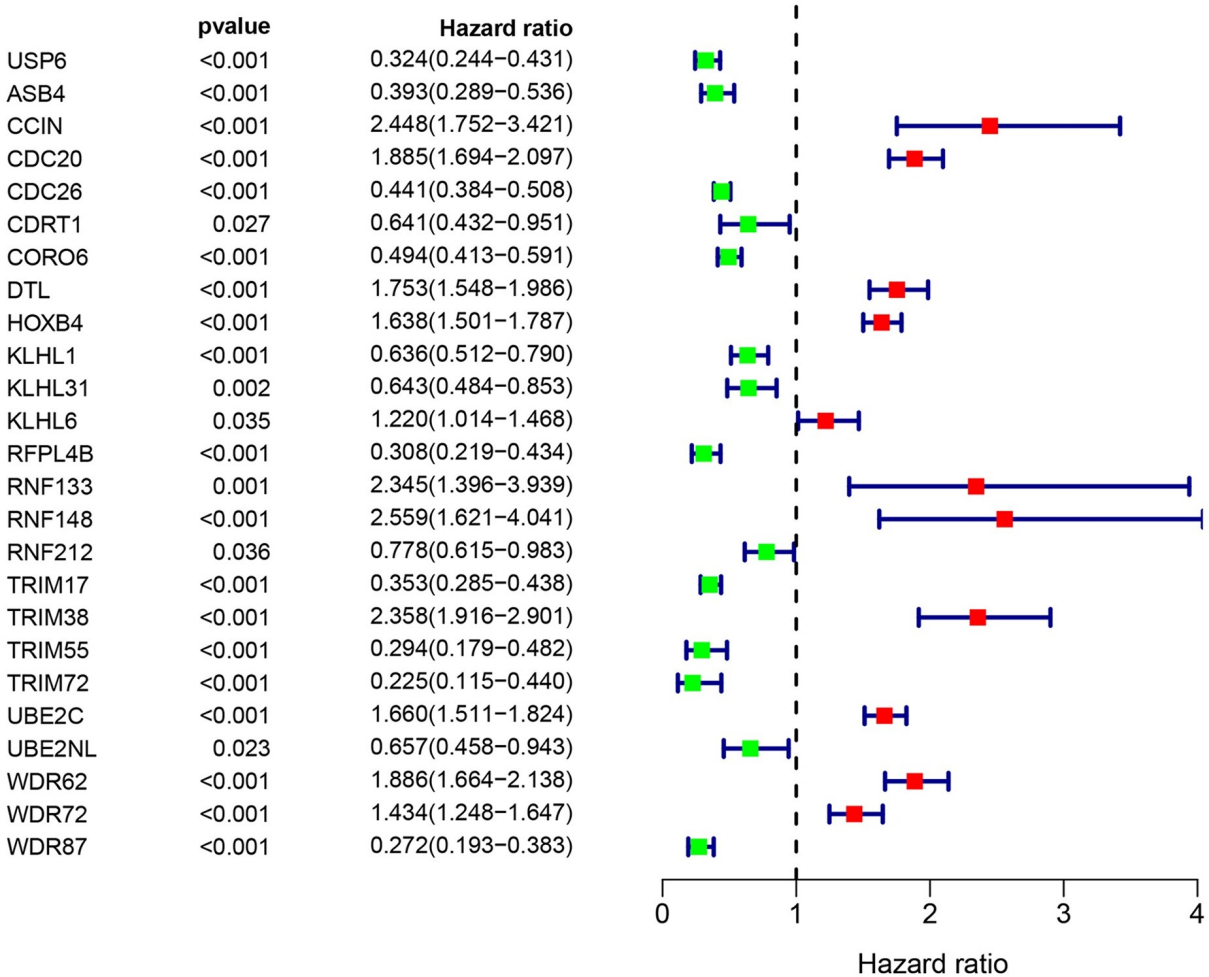
Identification of differentially expressed ubiquitination related genes (URGs) related to prognosis. Through joint analysis of TCGA (Including 697 specimens of glioma and 5 cases of non-tumor brain tissue) and GTEx (Containing 1152 healthy brain specimens before death) datasets, we identified 25 URGs related to survival. The line with a green dot indicates low risk factors, and the line with a red dot indicates high risk factors. P<0.05 are statistically significant.

**Fig 2:**
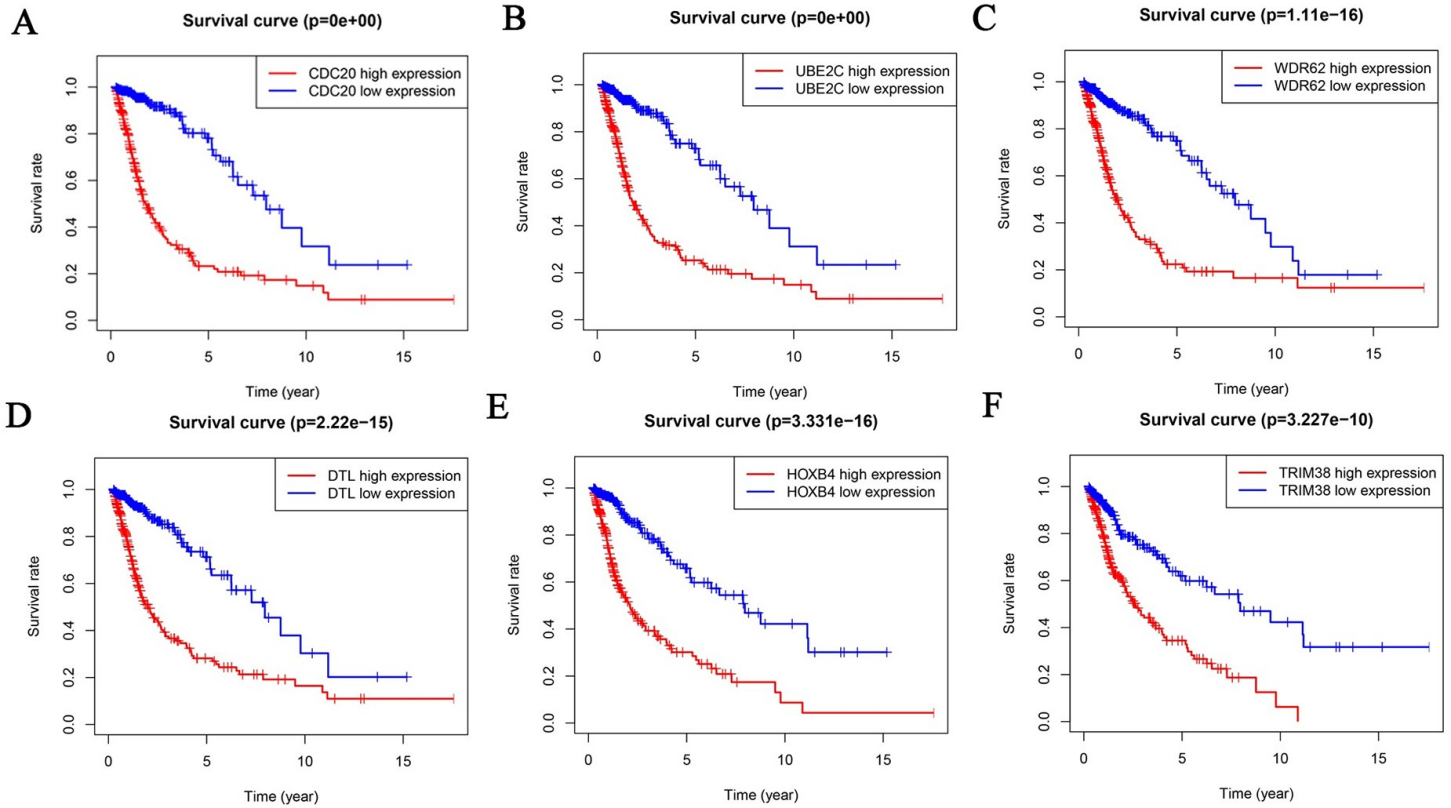
Survival curves of URGs related to the prognosis of patients. Survival analysis obtained URGs related to patient prognosis. Here we only showed the genes that also satisfy the true positive rate greater than 0.7 in ROC curve analysis: *CDC20* (A), *UBE2C* (B), *WDR62* (C), *DTL* (D), *HOXB4* (E) and *TRIM38* (F).

**Fig 3:**
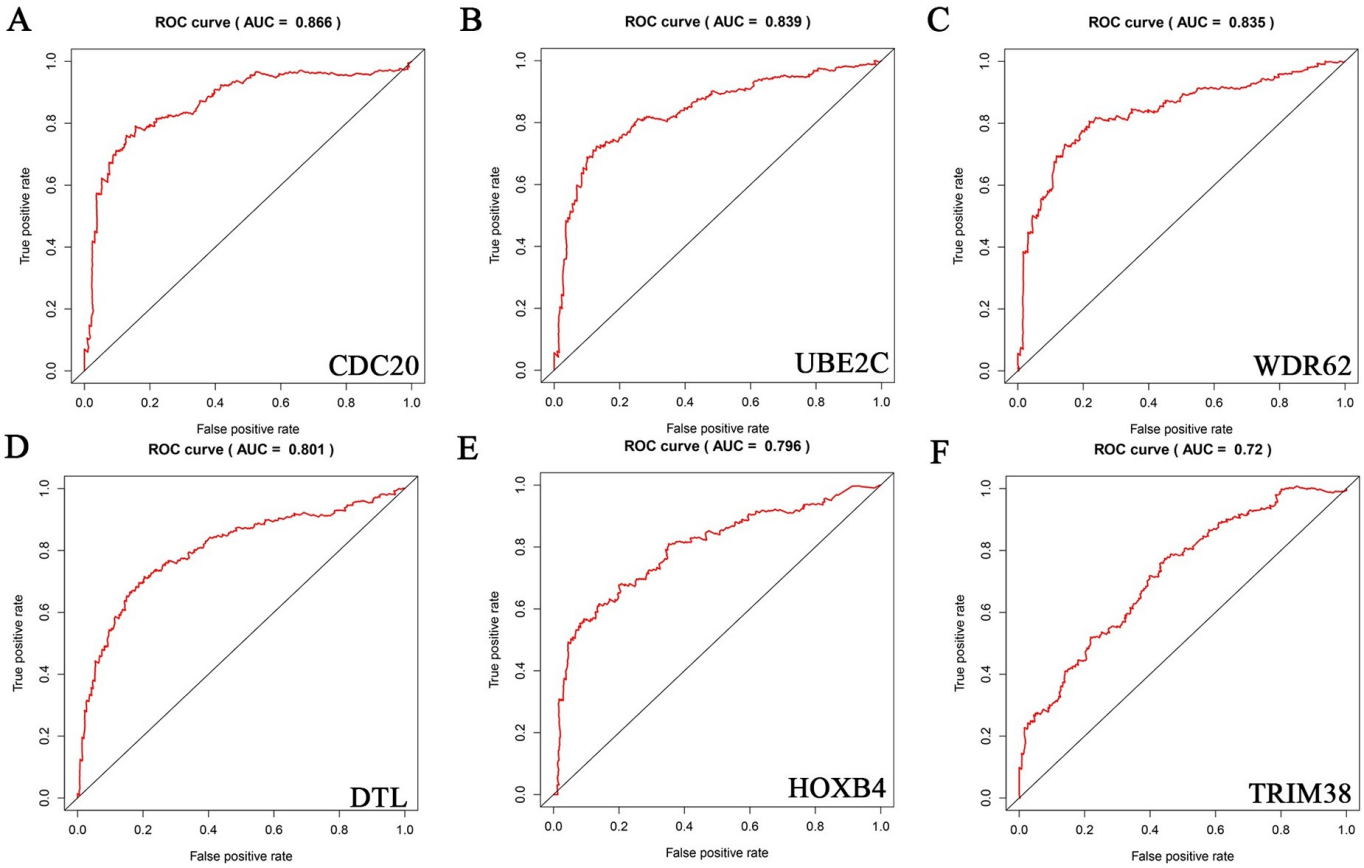
ROC curves for URGs related to patient prognosis. ROC curve analysis identified 6 genes with AUC greater than 0.7: *CDC20* (A), *UBE2C* (B), *WDR62* (C), *DTL* (D), *HOXB4* (E) and *TRIM38* (F).

**Fig 4:**
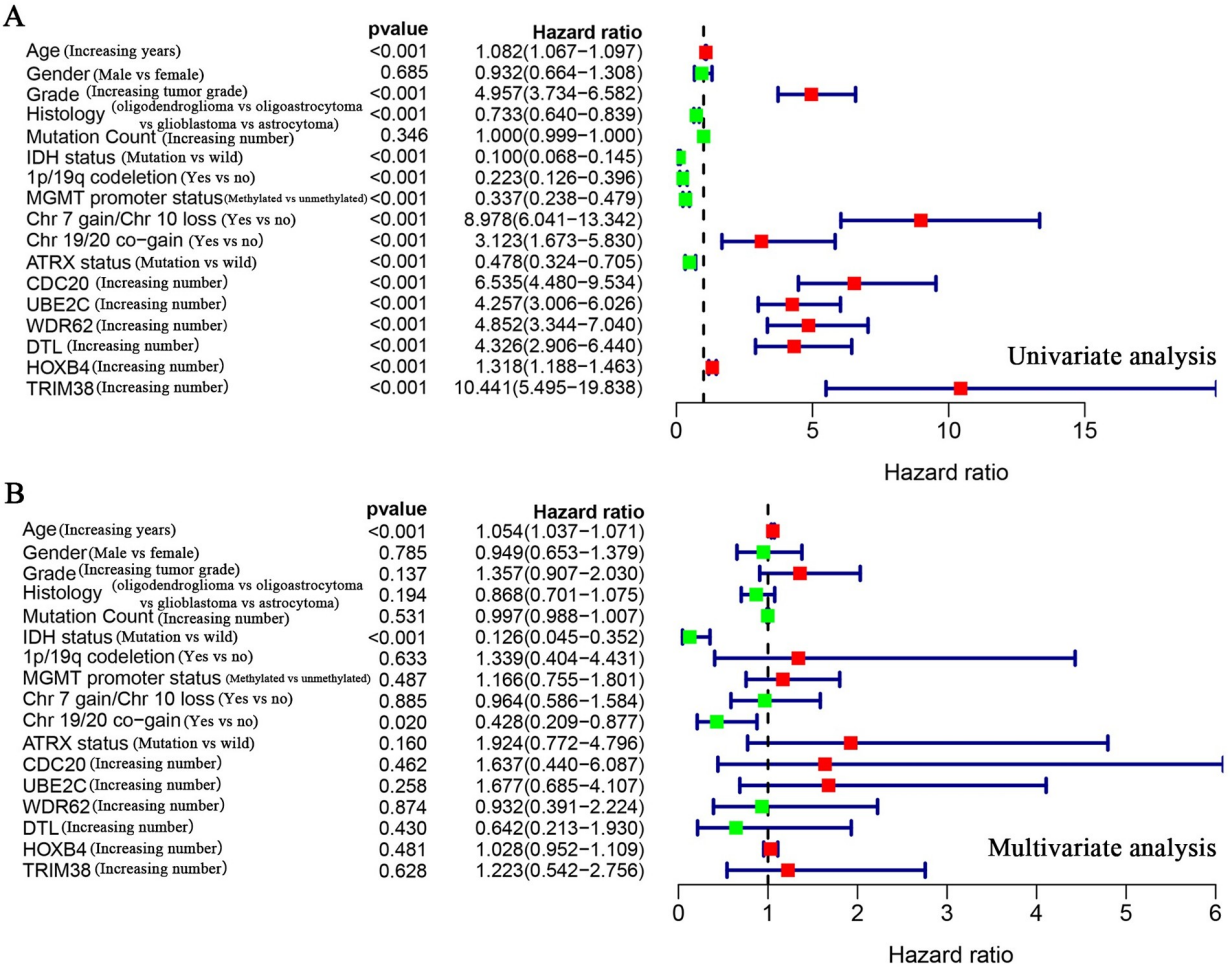
Cox analysis of the relationship of URGs with glioma patient prognosis. (A) Univariate Cox analysis found that except for gender and mutation count, they were all related to patient prognosis. (B) Multivariate Cox analysis found that only age, IDH status, and Chr 19/20 co-gain were independent factors for patient prognosis. The line with a green dot indicates low risk factors, and the line with a red dot indicates high risk factors.

By referring to the literature, we obtained ubiquitination-related genes (URGs), including 929 ubiquitin ligase genes and 95 deubiquitination enzyme genes. To determine whether URGs can be used as markers to judge the prognosis of glioma patients, we first obtained 36 differentially expressed URGs, including 19 downregulated genes and 17 upregulated genes, by comparing 1157 non-tumor brain tissues with 697 glioma tissues (Table 2). Cox analysis showed that 25 of these URGs were associated with patient survival (Fig 1). Among the 25 URGs, 11 were high-risk genes and 14 were low-risk genes. Survival analysis of *CDC20*, *UBE2C*, *WDR62*, *DTL*, *HOXB4* and *TRIM38* were statistically significant, and the AUC value was greater than 0.7 (Figs 2 and 3). We performed univariate and multivariate Cox analyses to determine the impact of individual genes on patient prognosis. Univariate analysis found that the six genes and more clinical traits (age, grade, histology, IDH status, 1p/19q co-deletion, MGMT promoter status, Chr 7 gain/Chr 10 loss, Chr 19/20 co-gain, ATRX status) are related to the prognosis of the patient without the influence of other factors (Fig 4A, Table 3). However, multivariate Cox analysis found that only age, IDH status and Chr 19/20 co-gain were independent factors for patient prognosis (Fig 4B, Table 3).

**Table 3:** Univariate analysis and multivariate analysis of the correlation of the expression of CDC20, UBE2C, WDR62, DTL, HOXB4 and TRIM38 with OS among glioma patients.

Parameter	Univariate analysis			Multivariate analysis		
	HR	95% CI	P	HR	95% CI	P
Age (Increasing years)	1.082	1.067−1.097	**2.12E-29**	1.054	1.037−1.071	**2.94E-10**
Gender (Male vs female)	0.932	0.664−1.308	0.685	0.949	0.653−1.379	0.785
Grade (Increasing tumor grade)	4.957	3.734−6.582	**1.76E-28**	1.357	0.907−2.030	0.137
Histology (oligodendroglioma vs oligoastrocytoma vs glioblastoma vs astrocytoma)	0.733	0.640−0.839	**6.89E-06**	0.868	0.701−1.075	0.194
Mutation Count (Increasing number)	1.000	0.999−1.000	0.346	0.997	0.988−1.007	0.531
IDH status (Mutation vs wild)	0.100	0.068−0.145	**4.17E-33**	0.126	0.045−0.352	**7.84E-05**
lp/19q co-deletion (Yes vs no)	0.223	0.126−0.396	**2.91E-07**	1.339	0.404−4.431	0.633
MGMT promoter status (Methylated vs unmethylated)	0.337	0.238−0.479	**1.20E-09**	1.166	0.755−1.801	0.487
Chr 7 gain/Chr 10 loss (Yes vs no)	8.978	6.041−13.342	**1.81E-27**	0.964	0.586−1.584	0.885
Chr 19/20 co-gain (Yes vs no)	3.123	1.673−5.830	**0.000**	0.428	0.209−0.877	**0.020**
ATRX status (Mutation vs wild)	0.478	0.324−0.705	**0.000**	1.924	0.772−4.796	0.160
CDC20 (lncreasing number)	6.535	4.480−9.534	**2.01E-22**	1.637	0.440−6.087	0.462
UBE2C (Increasing number)	4.257	3.006−6.026	**3.21E-16**	1.677	0.685−4.107	0.258
WDR62 (Increasing number)	4.852	3.344−7.040	**9.17E-17**	0.932	0.391−2.224	0.874
DTL (Increasing number)	4.326	2.906−6.440	**5.42E-13**	0.642	0.213−1.930	0.430
HOXB4 (Increasing number)	1.318	1.188−1.463	**2.09E-07**	1.028	0.952−1.109	0.481
TRIM38 (Increasing number)	10.441	5.495−19.838	**7.92E-13**	1.223	0.542−2.756	0.628

Bold values indicate P<0.05. HR, hazard ratio; OS, overall survival; CI, confidence interval.

### Construction of a clinical prognostic model for URG evaluation

**Fig 5:**
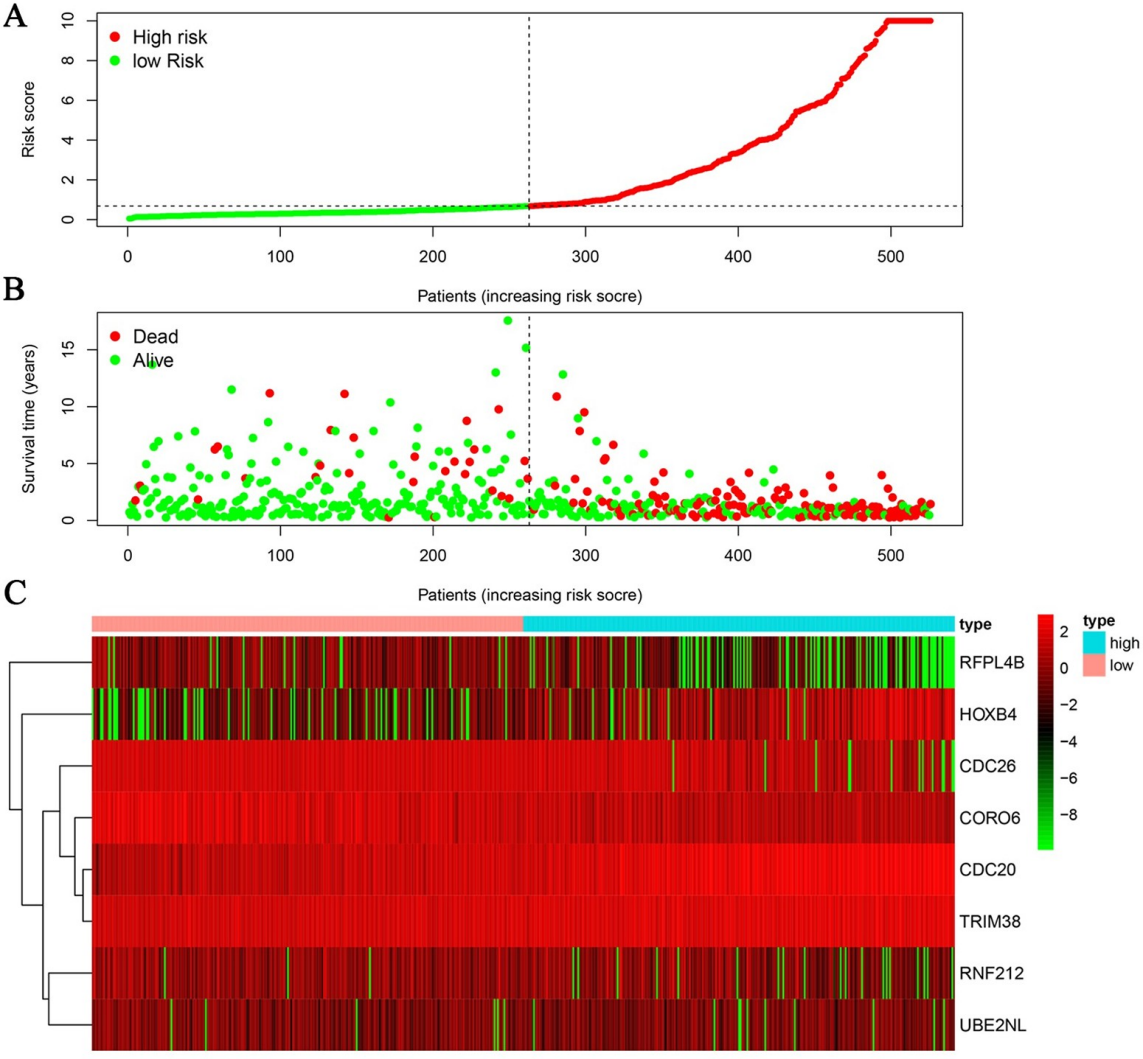
Patient prognosis based on the model constructed using URGs. (A) Patient prognosis risk score and distribution. (B) The survival time and status of patients with different risk scores. (C) Heatmap of URG expression profiles.

**Fig 6:**
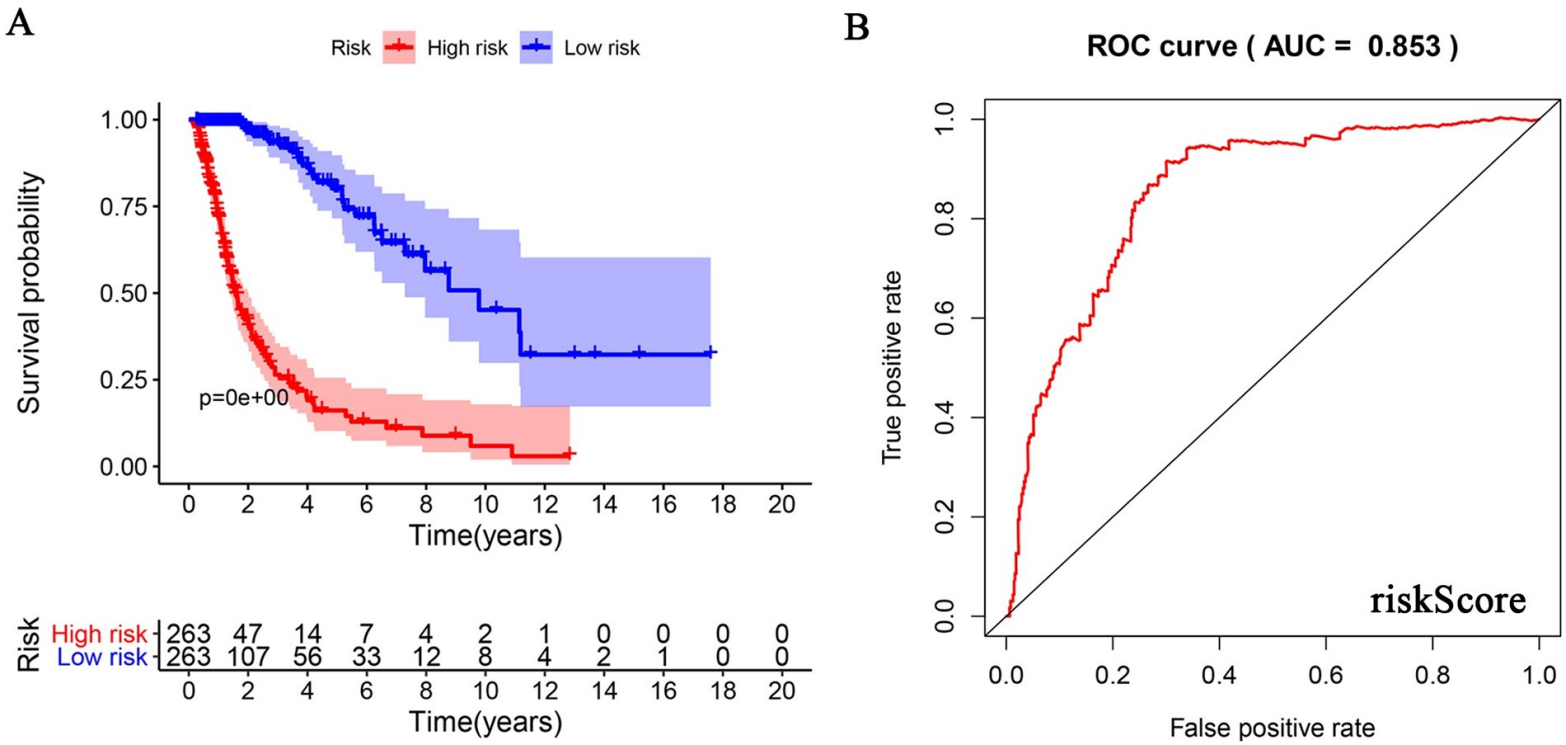
The prognostic value of the risk model. (A) Number of events and patient survival rate in high- and low-risk groups over time. (B) ROC curve analysis of the accuracy of the risk model. The true positive rate was 0.853.

**Fig 7:**
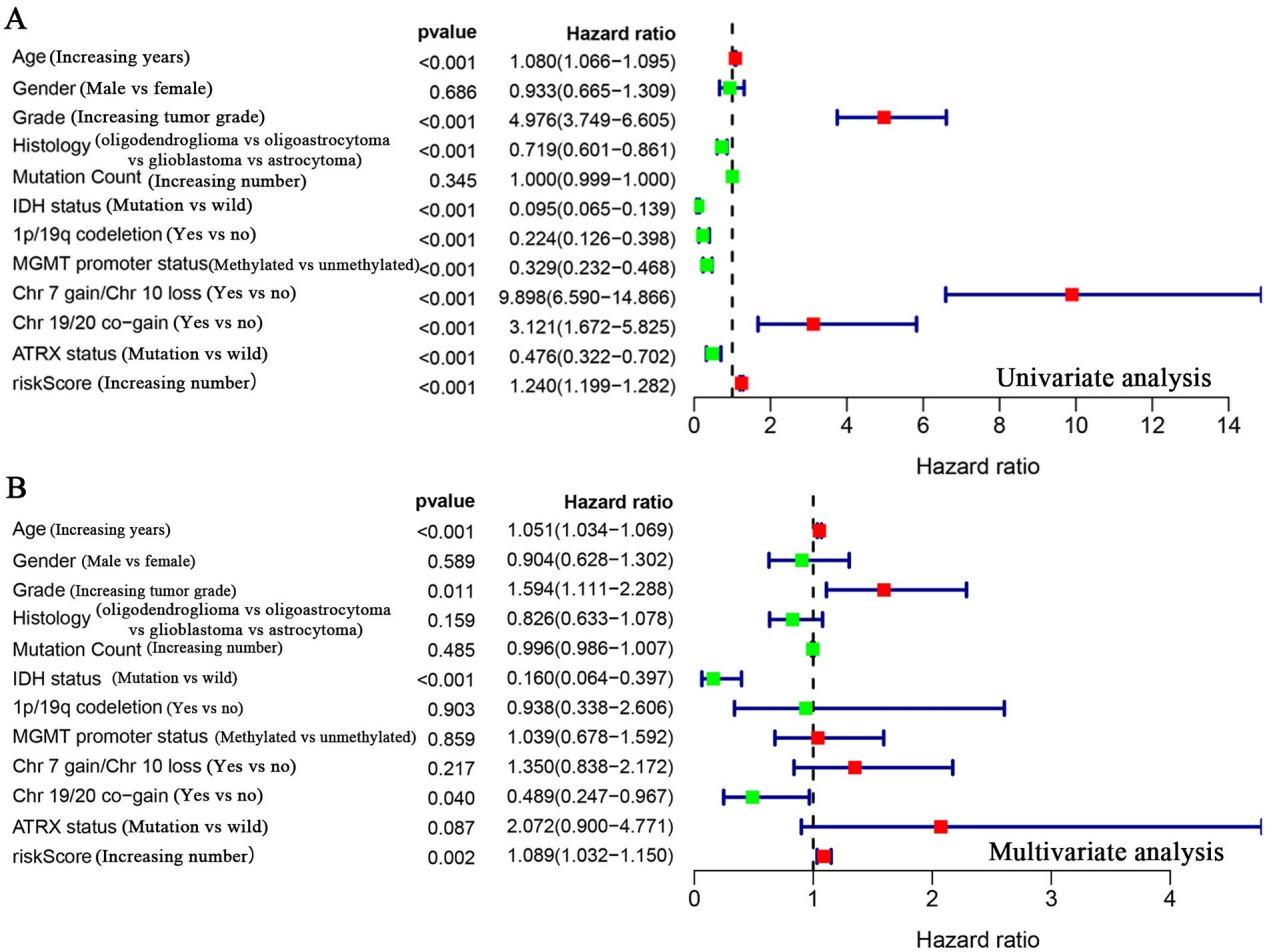
Analysis of risk model in predicting patient prognosis. (A) Univariate Cox analysis of the value of common clinical molecular markers and the risk model in predicting patient prognosis. Gender and mutation count showed no statistical significance with patient prognosis. (B) Multivariate Cox analysis of common clinical molecular markers and the risk model as independent prognostic factors. Age, grade, IDH status, Chr 19/20 co-gain and the risk model were independent prognostic factors. Parameters with green dots indicate low risk factors, and those with red dots indicate high risk factors.

Cox analysis found no independent prognostic factors among the survival-related URGs. Therefore, R software was used to analyze the relationship between the 25 differentially expressed URGs and the prognosis of patients and remove the URGs that can be correlated. We obtained an optimized risk model for predicting the prognosis of glioma patients (Table 4). Through multivariate Cox regression analysis, glioma patients were divided into high- and-low risk groups according to the median value of the risk value, and the risk value was calculated according to the risk model formula. A model was constructed to predict patient prognosis (Fig 5, Table 4). The formula is as follows: [*CDC20* expression level × (0.343) + *CDC26* expression level × (-0.308) + *CORO6* expression level × (-0.253) + *HOXB4* expression level × (0.173) + *RFPL4B* expression level × (-0.662) + *RNF212* expression level × (-0.361) + *TRIM38* expression level × (0.250) + *UBE2NL* expression level × (-0.381)]. Survival analysis showed that the 3-year and 5-year survival rates of patients in the high-risk group were 32.2% and 22.4%, respectively, and those in the low-risk group were 92.4% and 78.5%, respectively (Fig 6A). ROC curve analysis found that the model accuracy rate was 85.3% (Fig 6B). The model we constructed is expressed by riskScore, and univariate and multivariate Cox analyses showed that age, grade, IDH status, Chr 19/20 co-gain and riskScore can be used as independent prognostic factors (Fig 7, Table 5). In addition, we also constructed prognostic models for low-level and high-level groups, survival analysis and ROC analysis for verification in the TCGA and GSE108474 databases (Tables 6 and 7, S1 and S2 Figs).

**Table 4:** A model for predicting patient prognosis constructed with URGs.

Gene	coef	HR	HR.95L	HR.95H	pvalue
CDC20	0.343	1.410	1.222	1.626	2.40E-06
CDC26	-0.308	0.735	0.612	0.882	0.001
CORO6	-0.253	0.776	0.63	0.957	0.018
HOXB4	0.173	1.188	1.062	1.330	0.003
RFPL4B	-0.662	0.516	0.349	0.763	0.001
RNF212	-0.361	0.697	0.583	0.833	7.28E-05
TRIM38	0.250	1.285	0.986	1.673	0.063
UBE2NL	-0.381	0.683	0.466	1.002	0.051

Coef, coefficient; HR, hazard ratio; HR.95L, hazard ratio. 95% low; HR.95H, hazard ratio. 95% high.

**Table 5:** Univariate analysis and multivariate analysis of the correlation of the expression of riskScore with OS among glioma patients.

Gene	coef	HR	HR.95L	HR.95H	pvalue
ASB4	-1.915	1.473	0.022	0.972	0.047
CCIN	2.358	10.575	0.930	120.310	0.057
CDRT1	-1.376	0.253	0.045	1.408	0.116
DCAF4L2	-2.074	0.126	0.028	0.570	0.007
DTL	-5.875	0.003	0.000	0.050	6.58E-05
RNF148	-2.301	0.100	0.006	1.603	0.104
TRIM31	2.062	7.862	0.655	94.415	0.104
TRIM54	-2.249	0.106	0.025	0.448	0.002
WDR62	6.996	1092.750	38.060	31374.461	4.42E-05
WDR72	1.220	3.387	1.251	9.169	0.016

Coef, coefficient; HR, hazard ratio; HR.95L, hazard ratio. 95% low; HR.95H, hazard ratio. 95% high.

**Table 6:** A model for predicting grade II patient prognosis constructed with URGs.

Gene	coef	HR	HR.95L	HR.95H	pvalue
CCIN	0.283	1.328	0.909	1.940	0.142
CDC20	0.303	1.354	1.160	1.581	0.000
CDC26	-0.272	0.762	0.634	0.915	0.004
HOXB4	0.162	1.176	1.048	1.319	0.006
KLHL1	0.323	1.381	1.033	1.845	0.029
RNF212	-0.451	0.637	0.518	0.783	1.77E-05
TRIM17	-0.215	0.806	0.597	1.089	0.160
TRIM38	0.274	1.315	0.977	1.771	0.071
TRIM72	-1.062	0.346	0.139	0.859	0.022
UBE2NL	-0.467	0.626	0.419	0.938	0.023

Coef, coefficient; HR, hazard ratio; HR.95L, hazard ratio. 95% low; HR.95H, hazard ratio. 95% high.

**Table 7:** A model for predicting grade III and IV patient prognosis constructed with URGs.

Parameter	Univariate analysis			Multivariate analysis		
	HR	95% CI	P	HR	95% CI	P
Age (Increasing years)	1.080	1.066−1.095	**1.42E-28**	1.051	1.034−1.069	**1.69E-09**
Gender (Male vs female)	0.933	0.665−1.309	0.686	0.904	0.628−1.302	0.588
Grade (Increasing tumor grade)	4.976	3.749−6.605	**1.17E-28**	1.594	1.111−2.288	**0.011**
Histology (oligodendroglioma vs oligoastrocytoma vs glioblastoma vs astrocytoma)	0.719	0.601−0.861	**0.000**	0.826	0.633−1.078	0.159
Mutation Count (Increasing number)	1.000	0.999−1.000	0.345	0.996	0.986−1.007	0.485
IDH status (Mutation vs wild)	0.095	0.065−0.139	**4.70E-34**	0.160	0.064−0.397	**7.92E-05**
lp/19q co-deletion (Yes vs no)	0.224	0.126−0.398	**3.15E-07**	0.938	0.338−2.606	0.903
MGMT promoter status (Methylated vs unmethylated)	0.329	0.232−0.468	**5.73E-10**	1.039	0.678−1.592	0.859
Chr 7 gain/Chr 10 loss (Yes vs no)	9.898	6.590−14.866	**2.29E-28**	1.350	0.838−2.172	0.217
Chr 19/20 co-gain (Yes vs no)	3.121	1.672−5.825	**0.000**	0.489	0.247−0.967	**0.040**
ATRX status (Mutation vs wild)	0.476	0.322−0.702	**0.000**	2.072	0.900−4.771	0.087
riskScore (lncreasing number)	1.240	1.199−1.282	**5.56E-36**	1.089	1.032−1.150	**0.002**

Bold values indicate P<0.05. HR, hazard ratio; OS, overall survival; CI, confidence interval.

### The relationship between the risk model and clinical traits, signaling pathways, TFs and immune cells

**Fig 8:**
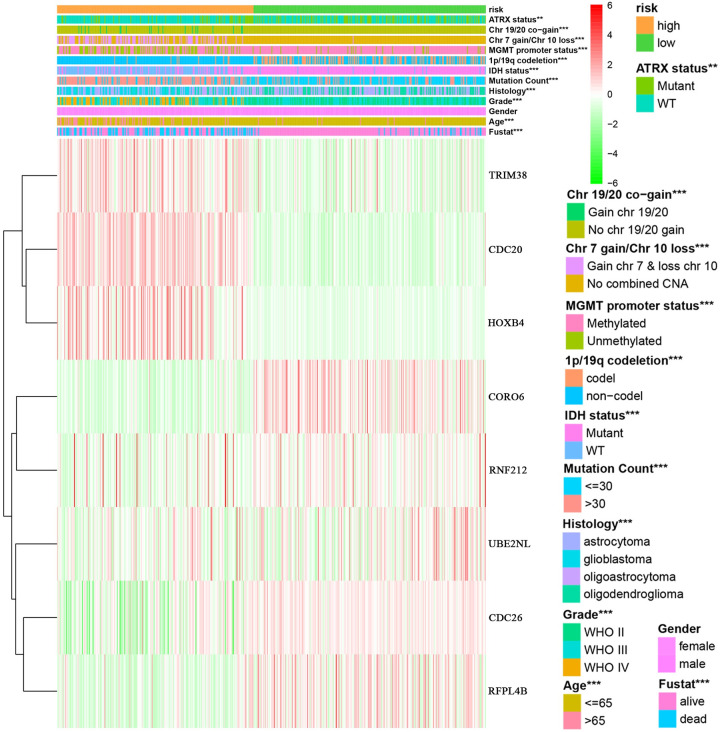
Relationship between risk groups and clinical traits. Analysis of the relationship between high and low risk groups and common clinical traits, including ATRX status, Chr 19/20 co-gain, Chr 7 gain/Chr 10 loss, MGMT promoter status, 1P/19q co-deletion, IDH status, mutation count, histology, grade, gender, age, and fustat. Gender and risk groups showed no correlation. TRIM38, CDC20, HOXB4 and other genes are the genes for constructing risk models. Red represents high expression, green represents low expression, and white represents intermediate expression. **p<0.01, ***p<0.001.

**Fig 9:**
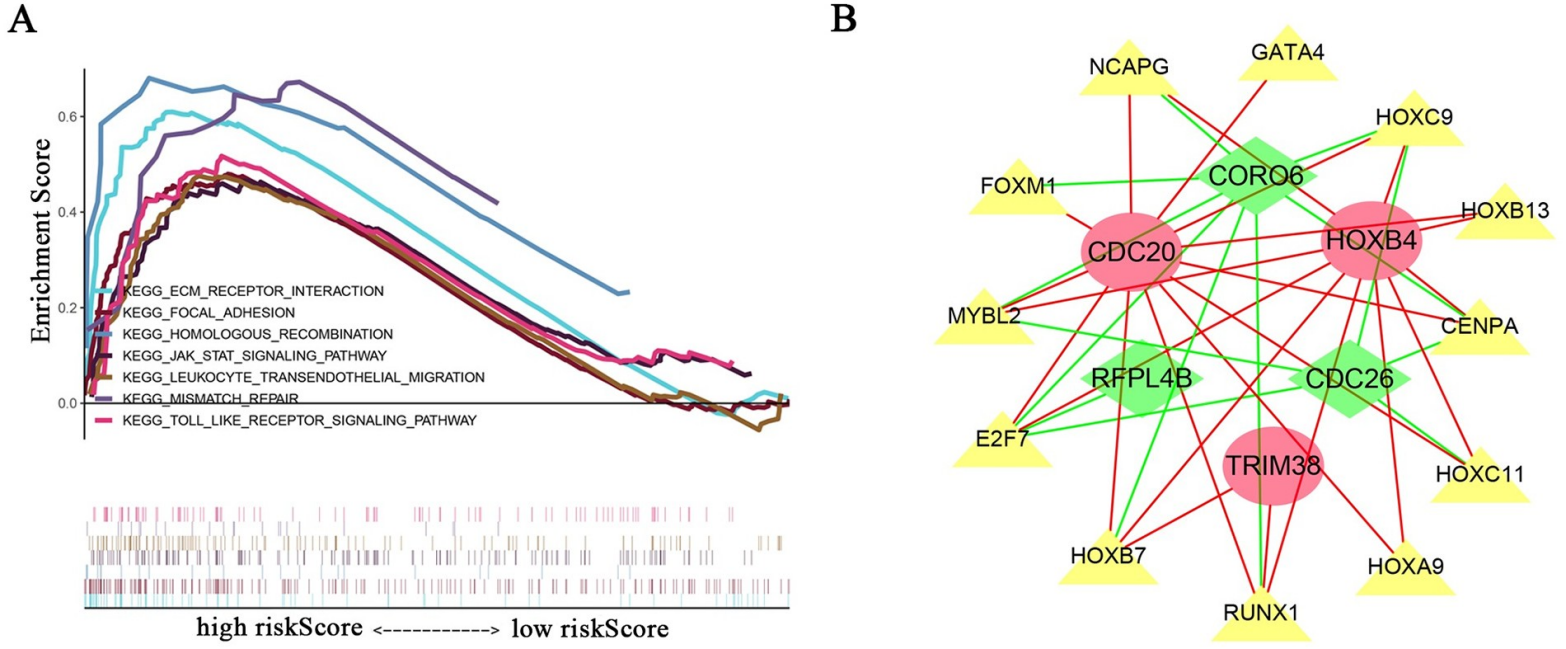
Enriched signaling pathways and TFs related to risk model genes. (A) GSEA enrichment analysis found that high-risk groups were enriched in ECM-receptor interaction, focal adhesion, homologous recombination, Jak-STAT signaling pathway, leukocyte transendothelial migration, mismatch repair, and the Toll-like receptor signaling pathway. (B) TFs that regulate risk model genes. Yellow triangles indicate TFs. Red ellipses and green quadrilaterals represent URGs; red ellipses indicate high-risk genes, and green quadrilaterals represent low-risk genes. The red line indicates a positive regulatory relationship, and the green line indicates a negative regulatory relationship.

**Fig 10:**
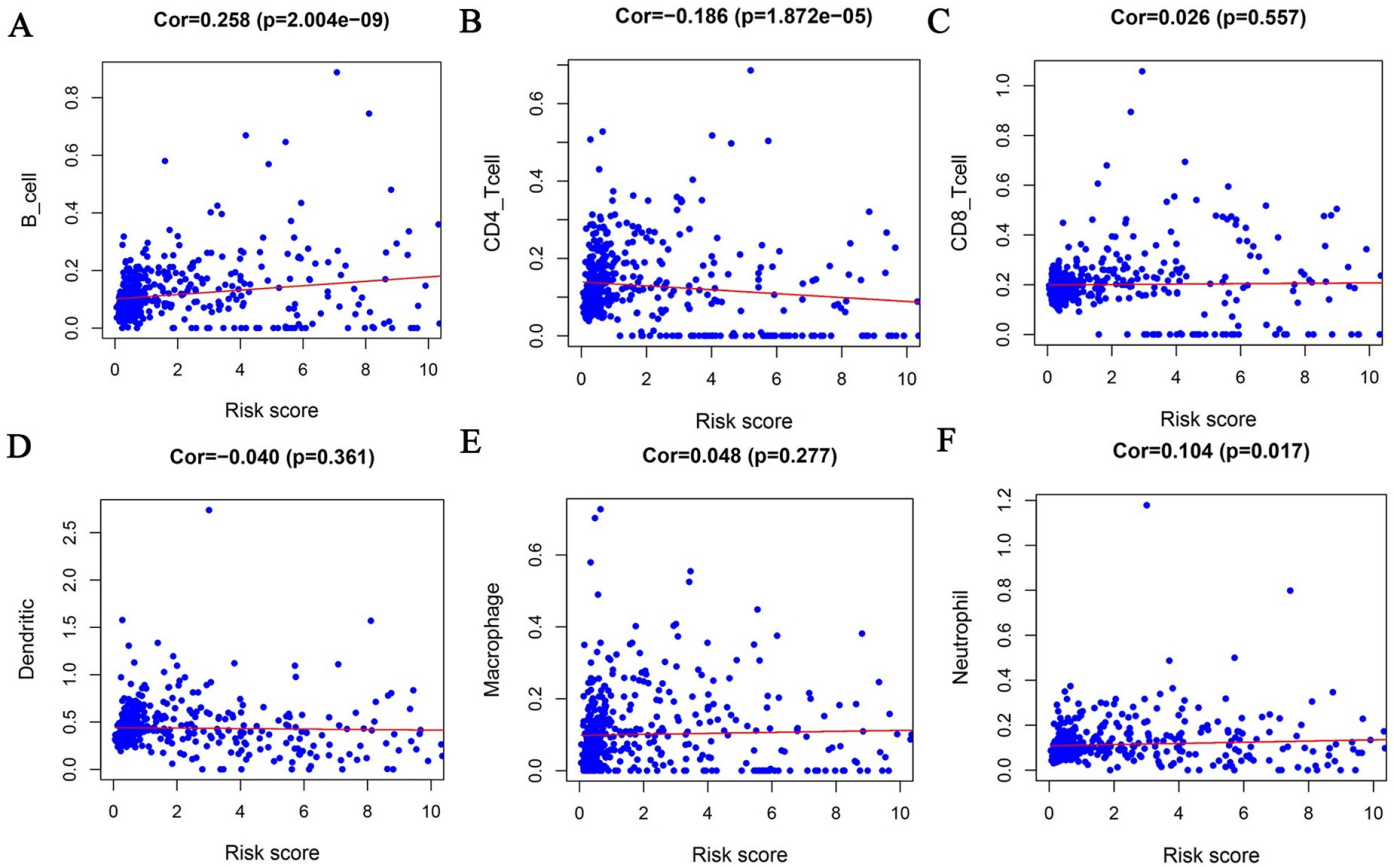
Relationship between risk score and immune cells. The risk score was related to B cells (A), CD4 T cells (B), and neutrophils (F). However, it showed no statistical correlation with CD8 T cells (C), dendritic cells (D) and macrophages (E).

We analyzed the relationship between the risk model and multiple clinical molecular markers and found that the risk model is related to ATRX status, Chr 19/20 co-gain, Chr 7 gain/Chr 10 loss, MGMT promoter status, 1P/19q co-deletion, IDH status, mutation count, histology, grade, and age (Fig 8). GSEA analysis revealed that the high-risk group is enriched in ECM-receptor interaction, focal adhesion, homologous recombination, Jak-STAT signaling pathway, leukocyte transendothelial migration, mismatch repair, and the Toll-like receptor signaling pathway (Fig 9A). However, no meaningful enrichment was found in the low-risk group. Cancer is caused by aberrant gene regulation. TFs are important molecules that directly regulate gene expression. To explore potential molecular mechanisms corresponding to the URGs in the risk model, we thus explored the potential TFs that may function in regulating the URGs in the risk model. Cytoscape was used to display TFs that regulate risk model genes (Fig 9B). GSEA analysis found that the risk score was related to leukocyte transendothelial migration. Since immune cell infiltration is very important in tumor progression, we next examined the relationship between the risk score and immune cells and found that it was related to B cells, CD4 T cells, and neutrophils (Fig 10).

## Discussion

By analyzing 929 ubiquitination ligase genes and 95 deubiquitination genes in the TCGA and GTEx databases, we obtained 36 differentially expressed URGs. Our results showed that 25 of the differentially expressed URGs were related to survival in glioma patients; six genes, *CDC20*, *UBE3C*, *WDR62*, *DTL*, *HOXB4*, and *TRIM38*, were statistically significant with an AUC value greater than 0.7. However, none of the six genes were independent prognostic factors for glioma patients. We constructed a model using the URGs that predicts the prognosis of glioma patients. Patients were stratified into high- and low-risk groups using the model, and the 5-year survival rates of the patients in the high- and low-risk groups were 22.4% and 78.5%, respectively, with a true positive rate of 0.853. Cox analysis showed that the risk model was independent factor for patient prognosis, in addition to age, grade, IDH status, and Chr 19/20 co-gain. The risk model was related to multiple factors such as ATRX status, Chr 19/20 co-gain, Chr 7 gain/Chr 10 loss, MGMT promoter status, 1P/19q co-deletion, IDH status, and mutation count, among other factors. GSEA found that the high-risk group was enriched in ECM-receptor interaction, focal adhesion, homologous recombination, Jak-STAT signaling pathway, leukocyte transendothelial migration, mismatch repair, and Toll-like receptor signaling pathway. In addition, we identified TFs that may regulate these genes and were related to B cells, CD4 T cells and neutrophils.

The degradation of intracellular proteins is mainly accomplished by the ubiquitin-proteasome system, and the ubiquitin-proteasome have strong selectivity for protein degradation [23]. Studies have shown that the ubiquitination pathway is closely related to the occurrence and development of tumors [1, 2, 8, 9, 19]. Here we identified six differentially expressed URGs that were related to survival in glioma patients. CDC20 drives the aggressiveness and self-renewal of gliomas and is associated with genomic instability [24, 25]. CDC26 is a component of the late cell cycle anaphase promotion complex, which is activated by CDC20 to regulate the cell cycle [26]. CORO6 is a member of the coronin family and is an actin-binding protein [27, 28]. HOXB4 is associated with the prognosis of ovarian cancer patients, leukemia resistance, and renal cancer [29–31]. RNF212 is associated with postoperative survival rate and TMZ chemoradiation response in GBM patients as well as mammalian meiosis [32, 33]. TRIM38 is associated with innate immunity and inflammation [34, 35]. Few studies have examined RFPL4B and UBE2NL. Our constructed model of URGs can be used as an independent prognostic factor to predict the prognosis of glioma patients.

With the completion of the Human Genome Project, some gene mutations have been shown to be aberrant in patients with gliomas and can be used for diagnosis and treatment of gliomas, such as IDH status, MGMT promoter status, and 1P/19q co-deletion etc [36–38]. We analyzed the relationship between the score of the risk model and these molecular markers of glioma and found that the risk score was closely related to the above molecular markers, which proves the reliability of the model. GSEA identified multiple tumor signaling pathways that may lead to disease progression in high-risk groups. Previous studies showed that the ubiquitination pathway regulates tumor progression through an immune response [1, 39]. GSEA found that the high-risk group was enriched for leukocyte transendothelial migration, and correlation analysis found that the risk score is related to the number of B cells, CD4 T cells and neutrophils.

In summary, we built a model of URGs to predict prognosis in glioma patients. GSEA results may have identified a possible mechanism in the patients with poor prognosis. Our results may provide new targets for the prognosis and treatment of glioma patients.

## Supporting information

S1 Table Curated ubiquitination-related genes lists. (XLSX)

S1 Fig Survival analysis and ROC analysis verification of high and low expression group. (TIF)

S2 Fig Test the reliability of the risk model in the GSE108474 database. (TIF)
