# Supplementary figures and images for: Identification of ubiquitination-related genes in human glioma as indicators of patient prognosis
Source: PLoS One. 2021 Apr 29;16(4):e0250239. doi: 10.1371/journal.pone.0250239 (PMC8084191; doi:10.1371/journal.pone.0250239)

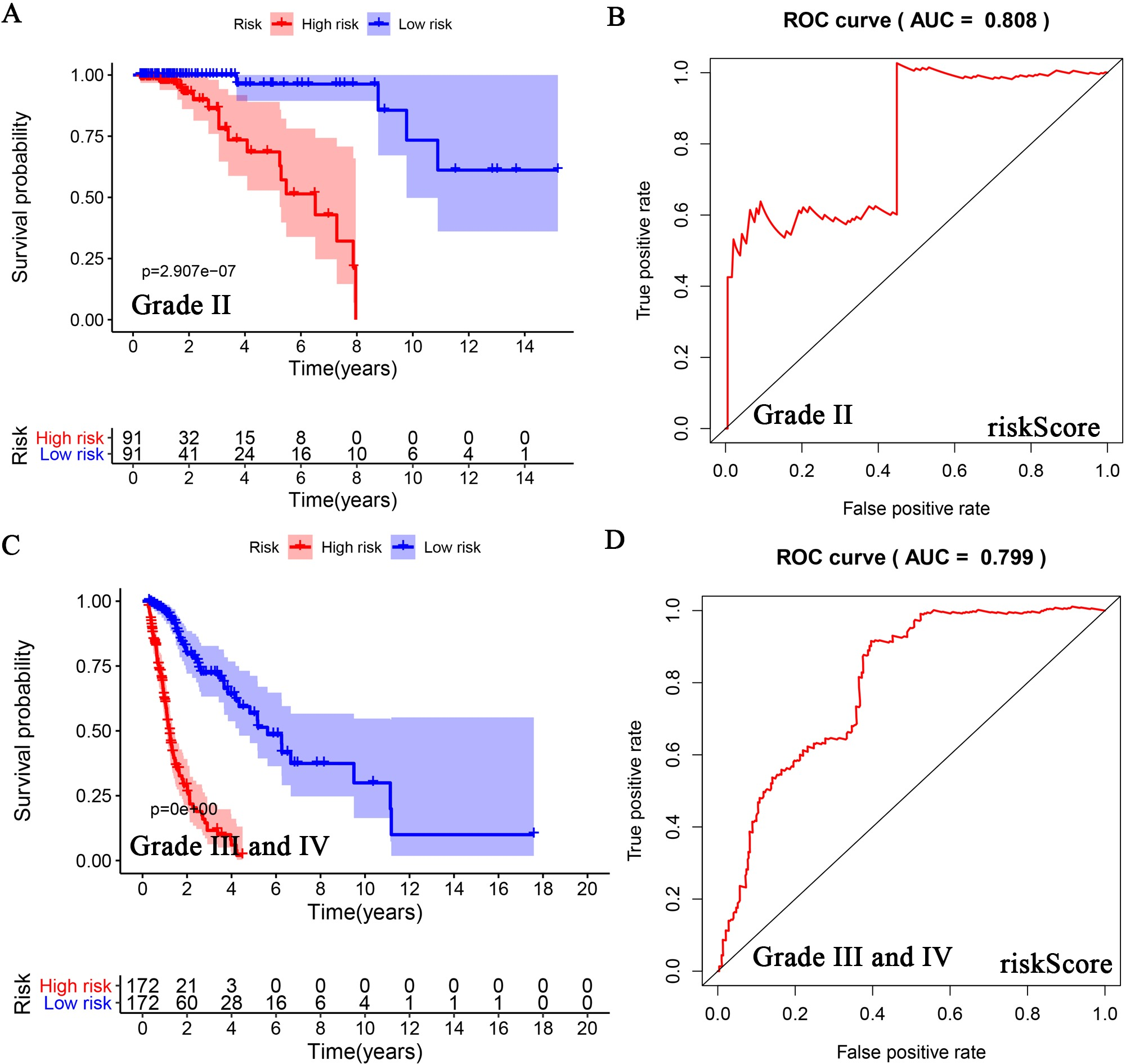

Supplement: S1 Fig — (TIF) [file pone.0250239.s002.tif]

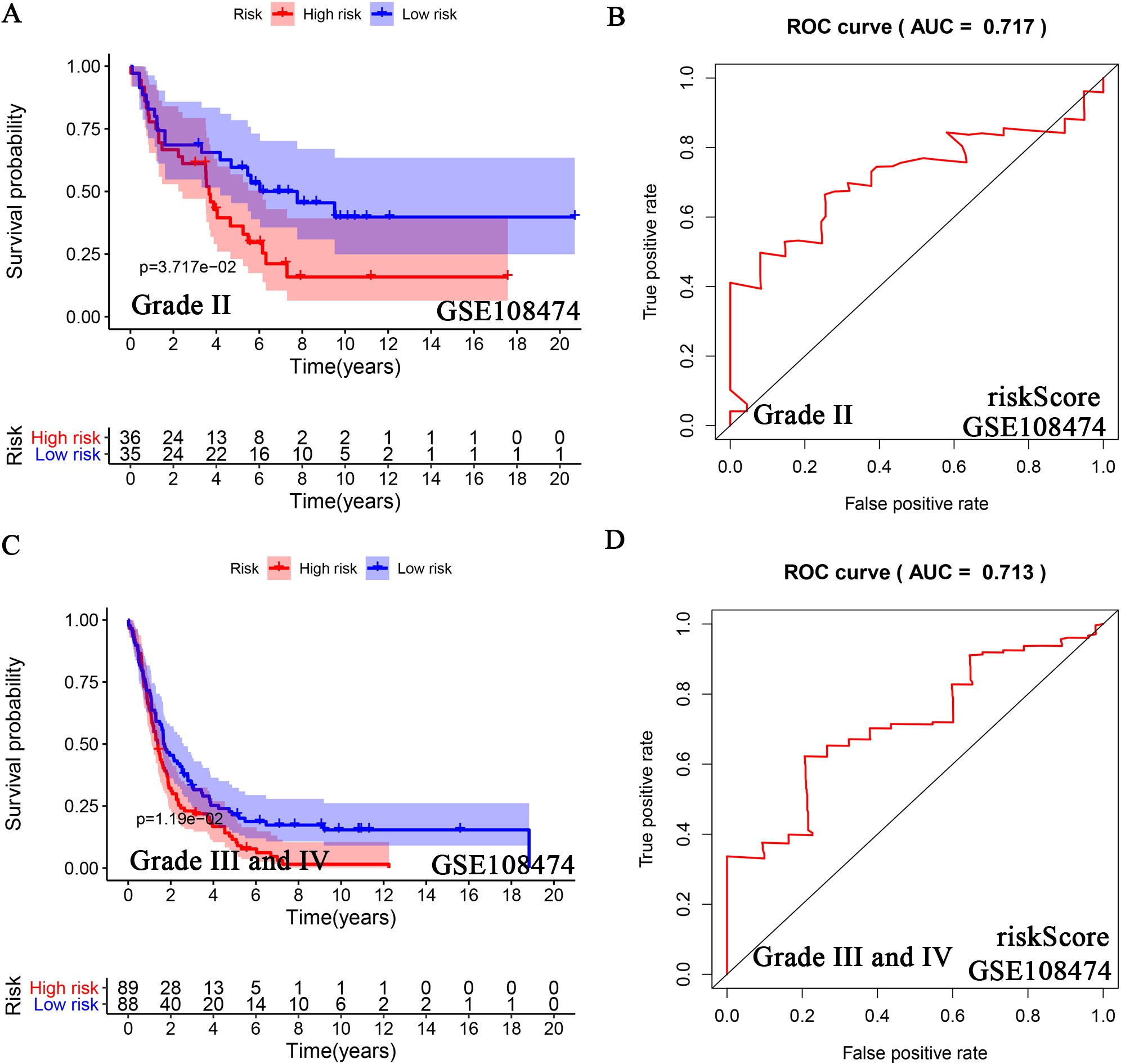

Supplement: S2 Fig — (TIF) [file pone.0250239.s003.tif]
